# The Function of Gastrointestinal Hormones in Obesity—Implications for the Regulation of Energy Intake

**DOI:** 10.3390/nu13061839

**Published:** 2021-05-27

**Authors:** Mona Farhadipour, Inge Depoortere

**Affiliations:** Translational Research in Gastrointestinal Disorders, Gut Peptide Research Lab, University of Leuven, Gasthuisberg, 3000 Leuven, Belgium; mona.farhadipour@kuleuven.be

**Keywords:** obesity, gastrointestinal hormones, nutrient sensing, circadian clock, gastric bypass surgery

## Abstract

The global burden of obesity and the challenges of prevention prompted researchers to investigate the mechanisms that control food intake. Food ingestion triggers several physiological responses in the digestive system, including the release of gastrointestinal hormones from enteroendocrine cells that are involved in appetite signalling. Disturbed regulation of gut hormone release may affect energy homeostasis and contribute to obesity. In this review, we summarize the changes that occur in the gut hormone balance during the pre- and postprandial state in obesity and the alterations in the diurnal dynamics of their plasma levels. We further discuss how obesity may affect nutrient sensors on enteroendocrine cells that sense the luminal content and provoke alterations in their secretory profile. Gastric bypass surgery elicits one of the most favorable metabolic outcomes in obese patients. We summarize the effect of different strategies to induce weight loss on gut enteroendocrine function. Although the mechanisms underlying obesity are not fully understood, restoring the gut hormone balance in obesity by targeting nutrient sensors or by combination therapy with gut peptide mimetics represents a novel strategy to ameliorate obesity.

## 1. Introduction

Obesity has increased dramatically over the past decades and reached epidemic proportions in adults and in children worldwide [1]. The rising prevalence and increased risk of developing chronic diseases exemplify the need for further research to improve understanding of the molecular mechanisms that are involved in the pathogenesis of obesity. Obesity is defined by the World Health Organization as “abnormal or excessive fat accumulation that may impair health” and is classified by a body mass index (BMI) ≥ 30 kg/m^2^, which is a simple index of weight-for-height [2]. Obesity reflects a high dietary intake relative to a low energy expenditure, which causes a disturbed energy balance [3]. However, obesity is a multi-factorial disorder and arises from the complex interaction between genetic, environmental, behavioral and psychological factors [4]. Genetic research has led to the recognition of rare monogenic and more common polygenic forms of obesity with different genes, each contributing to the relative risk of developing obesity [5]. This genetic predisposition is associated with genes that control eating behavior and appetite [6,7].

## 2. Regulation of Energy Homeostasis

The nuclei of the hypothalamus and brain stem play an important role in the regulation of energy homeostasis [8,9]. These central circuits integrate signals from the periphery to coordinate a response to a change in nutritional status. These signals act on two distinct populations in the arcuate nucleus that have projections to second order neuronal signalling pathways, which transform these inputs into behavioral responses to modify food intake and metabolic rate [9]. In general, peripheral signals can be divided into either long or short acting that communicate the energy status to the arcuate nucleus of the hypothalamus [10]. Long-term peripheral signals relay information about the extent of adipose tissue. These adiposity signals include the satiety hormone leptin, secreted by adipocytes, and insulin secreted by the pancreas. Their plasma levels are proportional to body fat and they can reach their receptors through an incomplete blood brain barrier at the level of the arcuate nucleus [11,12]. Short-term peripheral signals regulate energy homeostasis through the release of a number of peptide hormones that are secreted from enteroendocrine cells (EECs) in response to feeding and fasting [9]. In general, gut hormones are divided into hunger, orexigenic hormones and satiety, anorexigenic hormones. Ghrelin is the main hormone that is released from the stomach in response to fasting and triggers the onset of eating, thus regulating meal frequency [13]. There is also evidence that motilin is a hunger signal in humans through its stimulatory effect on gastric contractions in the fasted state that signal hunger via a cholinergic pathway [14,15]. The other hormones, including cholecystokinin (CCK), glucagon-like peptide 1 (GLP-1), gastric inhibitory peptide (GIP), peptide YY (PYY) and oxyntomodulin are satiety signals released after a meal and determine meal size [16,17,18]. However, a study in rats showed that non-nutrient driven GLP-1 release was possible prior to a meal after training the rats with time restricted feeding. The rats secreted GLP-1 cephalically in anticipation of a meal [19]. However, cephalic phase secretion in humans elicited by modified sham feeding was not observed for GLP-1 or ghrelin [20].

Brain regions may be activated either directly via the bloodstream or indirectly via activation of their receptors on the vagal nerve [9]. In addition, the vagus nerve induces satiety in response to nutrients through distension. This distention activates mechanoreceptors on intraganglionic laminar endings, which are present throughout the intestine, and contribute to stretch-induced meal termination [21,22]. Using single-cell RNA sequencing, Bai et al. provided a genetic map of vagal afferents innervating the gastrointestinal tract [23]. Food intake was most potently inhibited by vagal afferents that innervate the intestine. Stimulation of these mechanoreceptors activated satiety-promoting pathways in the brainstem to inhibit the hunger-promoting agouti-related protein (AgRP) neurons in the hypothalamus [23]. Furthermore, increasing intestinal volume was sufficient to inhibit food intake and AgRP activation even in the absence of nutrients.

In high-fat diet (HFD) induced obese mice, gastric and jejunal vagal afferents exhibit a reduced response to stretch which may lead to overconsumption of food and maintenance of the obese state [22].

## 3. Gut Hormones and Enteroendocrine Cell Plasticity

EECs begin as proliferating pluripotent stem cells in the crypt that commit to the secretory EEC lineage upon migration to the villi. EECs represent only 1% of the epithelial cell population [24]. There are several subtypes of EECs that have been classified based on their morphology and hormone production, e.g., P/D1-cells (ghrelin), L-cells (GLP-1, PYY, oxyntomodulin), I-cells (CCK), K-cells (GIP). However, the one cell-one hormone dogma was abandoned when it was shown, using flow cytometry and microarray-based transcriptomics, that some EEC subtypes were bi- or trihormonal and express members of functionally related peptides. It appeared that some EECs contain only 10% of their primary hormone (L-cells), while other EECs, such as the P/D1-cells, express more than 70% of their primary hormone, ghrelin [25,26]. This made the classification more complicated with up to 20 different cell types. These findings were further refined in a mouse single cell transcriptomic study which provided a clear description of the EEC hierarchy and its sub-lineages, identified regulators of lineage specification and showed that EECs display hormonal plasticity in the course of their maturation [27]. Recently, Beumer et al. provided a high-resolution mRNA and secretome atlas of human EECs using an organoid-based platform [28]. Key differences to murine EECs were found with respect to hormones, sensory receptors and transcription factors. For example, although motilin EECs do not exist in mice, in humans they identified a cluster of cells producing motilin and ghrelin with a gradient from predominantly motilin to mainly ghrelin expressing cells [29]. The authors speculated that these might represent different states of the same cell type that can undergo a bone morphogenic protein (BMP)-controlled switch in hormone expression [28]. Another study from this group showed that upon activation of BMP signalling L-cells lose GLP-1 and increase secretin, neurotensin and PYY expression [30]. Therefore, BMP inhibitors have the potential to boost GLP-1 producing cells to improve glycaemic control in the management of type 2 diabetes mellitus (T2DM) [30].

## 4. Altered Gut Hormone Levels in Obesity

Obesity is known to affect the expression of many hormones. Defects in the long-acting peripheral signals can be due to mutations in the leptin gene or leptin receptor, and have been observed in a limited number of patients [31]. Leptin deficiency is entirely treatable with daily subcutaneous injections of recombinant human leptin. However, the majority of obese patients have high circulating leptin levels and in these patients administration of recombinant leptin failed to decrease body weight and food intake due to leptin resistance [32]. The latter contributes to the maintenance of obesity. Several mechanisms have been identified as potentially underlying leptin resistance, including changes in transport across the blood brain barrier, endoplasmatic reticulum stress and impaired leptin receptor function and STAT3 signalling [33]. A recent study in aged mice even showed that the leptin sensitizer, celastrol, restored sensitivity and induced decreases in fat mass and body weight, but not in young mice [34]. In addition, the vagal afferent pathway plays an important role in signalling of the nutrient content from the gut to the brain through sensing of the gut hormones [35]. De Lartigue et al. found that leptin resistance by the vagal afferent pathway causes hyperphagia, which contributes to the onset of obesity [36]. The sensitivity of gastric vagal afferents that convey satiety signals is decreased in HFD-induced obese mice. In contrast to lean mice, leptin released from gastric epithelial cells further inhibited the response of vagal afferents to mechanical stimuli in HFD-induced obese mice, thereby worsening the situation [37].

Obesity also alters the short-term meal-related signalling of gut hormones. It is tempting to speculate that changes in the gut hormone balance may contribute to hyperphagia in obese patients. Table 1 summarizes the meal-related fluctuations in gut hormone levels and their alterations in obese and T2DM patients.

### 4.1. Orexigenic Hormones

#### 4.1.1. Ghrelin

Ghrelin is a 28-amino acid peptide that is activated upon octanoylation of serine on the third N-terminal amino acid position, a posttranslational modification initiated by ghrelin O-acyl-transferase (GOAT) [59,60]. Ghrelin binds to the growth hormone secretagogue receptor (GHSR1a) on NPY/AgRP neurons in the arcuate nucleus of the hypothalamus to stimulate food intake [61]. However, ghrelin also stimulates many brain regions that are involved in reward and motivation, including the ventral tegmental area and hippocampus [62]. Ghrelin favors hedonic food consumption by enhancing reward signalling [62,63,64].

Ghrelin is secreted from the P/D1 cells during the preprandial state and influences the frequency of meals [13]. Secretion of ghrelin involves the sympathetic nervous system and is mediated via β1 receptors on the ghrelin cell [65,66]. The magnitude of the postprandial fall is dependent on the macronutrient composition of the meal and caloric content [67,68]. Plasma ghrelin levels are lower in obese patients [38,39,40,41,42,43]. This decrease is thought to be compensatory rather than causal and to represent a physiological adaptation to the positive energy balance. The only exception is patients with Prader–Willi syndrome, who are characterized by high ghrelin levels long before the onset of hyperphagia [69]. At an early age, these patients suffer from persistent cravings, which lead to increased food intake and childhood obesity. In addition, these patients not only show endocrine dysfunctions such as growth hormone deficiency hypogonadism, hypothyroidism and several others, but also behavior problems, social/learning disabilities, mental issues and comorbidities [70].

Furthermore, obese patients have almost no postprandial decline in response to food intake, and the lack of meal-related fluctuations may continuously stimulate appetite [39]. There are inconsistent findings on the effect of obesity on the number of ghrelin-positive cells and ghrelin mRNA expression in biopsies [40,71,72]. In resection specimens, region-dependent differences in ghrelin mRNA expression were observed in the stomach of lean versus obese subjects, with higher expression levels in the distal stomach of obese subjects compared to lean [73]. Studies in human primary fundic cultures revealed that obesity decreased the production of ghrelin at the protein level in the cell, resulting in a decreased secretion in the cell supernatant without affecting steady state secretory mechanisms [74]. Altered responsiveness to noradrenaline has been observed in primary crypt cultures from obese subjects, as well as from diet-induced mice, suggesting that a reduced sympathetic drive may contribute to the disturbed ghrelin regulation [74,75].

Studies in diet-induced obese (DIO) mice suggest that the hypothalamic circuitry, which controls food intake, becomes resistant to ghrelin during obesity [76]. Several mechanisms have been suggested, including hyperleptinemia and inflammation in the hypothalamus and nodose ganglion. Recently, it has been hypothesized that high levels of liver-enriched antimicrobial peptide-2, the endogenous ghrelin receptor antagonist produced in the small intestine, prevent acylated ghrelin from activating ghrelin receptors in the arcuate nucleus in obese individuals [77].

Obese patients may potentially only benefit from treatments with GHSR antagonists or treatments that neutralize ghrelin (e.g., GOAT inhibitors) after diet-induced weight loss, which restores ghrelin sensitivity and is accompanied by an increase in plasma ghrelin levels. Nevertheless, low dose infusion of ghrelin still increased ad libitum energy intake at a buffet meal in obese patients [78]. Therefore, the concept of ghrelin resistance remains to be shown in obese patients.

#### 4.1.2. Motilin

Motilin is a 22-amino acid peptide released by the M-cells in the small intestine that stimulates gastrointestinal motility [79]. It is structurally related to ghrelin, which was originally named motilin-related peptide [80]. Plasma motilin levels fluctuate with the phases of the migrating motor complex (MMC), initiating in the distal stomach or small intestine a pattern of strong contractions during the interdigestive phase that clean the intestine of food remnants [81]. More recently, motilin was identified as a key hunger signal in humans. It was shown that motilin-induced gastric phase III contractions during the MMC signal hunger in the fasting state via a cholinergic pathway [14,15].

Deloose et al. reported higher plasma motilin levels with less fluctuations in the fasting state in obese patients [44]. The lack of elevation in plasma motilin levels before the start of gastric phase III contractions was in line with the switch in origin of phase III contractions from the stomach to the duodenum, initially described by Pieramico et al. [82] This may explain the reduced hunger observed in obese patients during phase III that was restored by pharmacological induction of gastric phase III contractions with the motilin receptor agonist, erythromycin [44].

### 4.2. Anorexigenic Hormones

#### 4.2.1. Cholecystokinin

Cholecystokinin (CCK) is secreted mainly by duodenal and jejunal I-cells in response to feeding, particularly fat and protein [83]. It is posttranslationally processed to yield various truncated circulating forms including CCK-8, CCK-33 and CCK-58, that can bind to the CCK1 receptor or CCK2 receptor to exert its biological effects. CCK inhibits food intake by acting on vagal afferents, especially in the duodenum [84]. Slowing of gastric emptying is another mechanism of CCK-induced appetite suppression [85]. In pharmacological concentrations, CCK also stimulates insulin secretion [86].

It remains controversial whether obesity affects CCK secretion; both a reduction [43,45], an increase [46] and unaltered [47] postprandial CCK levels have been reported in obese subjects. Furthermore, the responses to macronutrients have been variable [47]. Defects in CCK signalling have been reported to contribute to obesity, since genetic mutations in CCK1 receptor result in increased meal size and food intake [87,88].

Obese humans are still sensitive to the satiating action of CCK [89]. However, in humans the efficacy of molecule CCK1 receptor agonists has been variable [90]. Furthermore, these molecules suffer from lack of specificity and have off-target effects. Novel strategies are needed to target CCK1 receptors more effectively and to dissociate disease activity from undesirable effects [91].

#### 4.2.2. Glucagon-Like-Peptide 1

Glucagon-like peptide-1 (GLP-1) is a proglucagon-derived hormone that is secreted by L-cells in the small intestine and colon in response to nutrients [16]. L-cells are in direct contact with luminal nutrients and GLP-1 levels increase rapidly upon food intake. The low number of L-cells in the proximal intestine probably accounts, at least in part, for the early postprandial rise in GLP-1 levels, but it has been suggested that neuronal and/or humoral mechanisms contribute as well. GLP-1 is a satiety signal that mainly acts via vagal, rather than, central GLP-1 receptors [16,92]. Once GLP-1 is taken up by capillaries, it is rapidly broken down by dipeptidyl peptidase (DPP4). This limits the amount of GLP-1 reaching the systemic circulation and hence, its endocrine activities. GLP-1 is an important incretin hormone that stimulates glucose-dependent insulin secretion by binding to GLP-1 receptors on β-cells [93].

A study in a large cohort of obese patients showed a ~20% reduction in GLP-1 response to oral glucose compared with normal weight individuals [48]. However, when comparing GLP-1 secretion during an oral glucose tolerance test or meal-tolerance test in subjects with obesity or T2DM, increased [53], decreased [48,49,50,51,52] or unchanged [54,55,56] GLP-1 responses have been found. In some studies, patients with T2DM were treated with metformin or DPP4 inhibitors, which enhance GLP-1 secretion, and in others insulin resistance was more pronounced, a factor related to impaired GLP-1 release [94]. If the cohorts are not matched closely to the healthy participants, factors such as age, sex and rate of gastric emptying, may all influence the secretion of GLP-1.

Indeed, the pathology of obesity may change according to age and gender, and therefore affect the changes in the levels of the gut hormones. For example, pre-menopausal women may have higher GLP-1 levels relative to their control groups (post-menopausal women and men of the same age), which may provide premenopausal women with relative protection against metabolic diseases and the associated comorbidities [95,96].

Since reduced GLP-1 levels are not representative for all patients, it remains to be further investigated in longitudinal studies whether a decrease in intestinal GLP-1 secretion contributes to obesity in humans. Studies in animal models are also inconsistent with reports of higher GLP-1 levels in diet-induced obese rats than in control rats [97].

Nevertheless, although obesity may affect postprandial GLP-1 secretion, obese patients are still sensitive to systematically administered GLP-1 with consequent reduced hunger ratings and slowed gastric emptying. In fact, GLP-1 receptor agonists and DPP4 inhibitors are widely used classes of anti-diabetic and/or anti-obesity agents [98,99].

#### 4.2.3. Peptide YY

PYY is a 36-amidated amino acid peptide that is secreted by L-cells in the distal gut together with GLP-1, GLP-2 and oxyntomodulin following a meal [100]. Proteins provide the most potent stimuli for the release of PYY [57]. Immediately after an oral nutrient load, PYY levels start to rise even before the nutrients reach the distal gut, implying the involvement of a neural reflex pathway [101]. PYY_1-36_ is cleaved to PYY_3-36_ by the enzyme DPP4 immediately after secretion. PYY_3-36_ acts at Y2 receptors in the arcuate nucleus to inhibit food intake [102]. Meal-induced PYY_3-36_ release tends to be lower in obese than in lean individuals [43,57,58]. Whether peripheral PYY_3-36_ acts as a satiety signal in rodents remains controversial [102,103]. After the initial report by Batterham et al. that peripheral injection of PYY_3-36_ inhibited food intake and reduced body weight in mice but not in Y2R-null mice, several other labs (data from 1000 rodents obtained in 12 labs) failed to replicate these findings [103,104]. Stress was suggested to be a confounding factor. Nevertheless, a follow-up report showed the PYY_3-36_ reduced appetite by 30% in 12 obese and 12 lean volunteers [58].

There are currently two PYY_3-36_ compounds, PYY 1875 (Novo Nordisk) and GT-001 (Gila Therapeutics) in phase 1 trials for the treatment of obesity.

## 5. Altered Nutrient Sensing in Obesity

Nutrient-sensing G-protein-coupled receptors (GPCRs), similar to those in the lingual system, are present on epithelial cells in the gut and respond to luminal compounds (nutrients, bile acids, bacterial metabolites, toxins etc.) to induce a variety of biological functions [105,106]. Chemoreceptors on EECs in the gut tune the balance of appetite-regulating hormones in response to a meal. Both in vitro studies (cell lines, mucosal segments and isolated crypts) and in vivo studies (taste receptor knockout mice) have been used to demonstrate a role for taste receptors in gut hormone release [106]. For example, in the stomach protein breakdown products are sensed by the umami receptor (TAS1R1–TAS1R3) and other amino acid sensors (CaSR and GPRC6A) on P/D1-cells, to regulate the release of ghrelin [73,107]. Carbohydrate sensing (TAS1R2-TAS1R3, Na^+^-glucose cotransporter type 1 (SGLT1)) occurs mainly in L-cells which secrete GLP-1 [108,109]. In addition, bitter taste receptors on EECs may represent an important target to reduce appetite [110].

The nutrient-sensing mechanisms of EECs may be affected by obesity and influence meal-related gut hormone fluctuations. The expression of amino acid sensors and bitter taste receptors in the mucosa of the human stomach and small intestine is affected by obesity in a region-dependent manner [73,74]. Similar findings were reported in HFD induced obese mice [111]. The effect of a casein hydrolysate on ghrelin release was reduced in mucosal segments of the human fundus, whereas the effect of the broadly tuned bitter agonist denatonium benzoate was apparently selectively blunted by obesity in human small intestinal—but not in fundic— segments [73,74]. Nguyen et al. showed that in morbid obesity, proximal intestinal glucose absorption is accelerated and related to increased expression of SGLT1, and may predispose to T2DM [112]. In patients with T2DM the expression of sweet taste receptors has proven to be dysregulated during acute hyperglycemia, and this may contribute to postprandial hyperglycemia [113]. Stewart et al. showed that the sensing of oleic acid by lingual and also intestinal receptors is compromised in obese patients [45]. Furthermore, the increase in plasma CCK levels in response to oleic acid tended to be reduced in the obese population.

A loss of function mutation has been observed in the long chain fatty acid receptor, FFAR4, in obese individuals that increased the risk of obesity and T2DM [114].

Thus, changes in the nutrient sensing mechanisms of EECs have been observed in obesity. The effects are region- and nutrient-specific and may, therefore, also be influenced by the diet of the obese patients. This may contribute to some of the inconsistent findings related to the effect of obesity on meal-related fluctuations in gut hormone levels.

## 6. Obesity Alters the Circadian Clock and the Diurnal Fluctuations in Gut Hormone Levels

Apart from meal-related fluctuations, gut hormones also show diurnal fluctuations that are regulated by the circadian clock in the hypothalamic suprachiasmatic nucleus. The most important entrainment signal of the master clock in mammals is the light–dark cycle, which inevitably determines the feeding–fasting cycle, which in turn indirectly entrains peripheral clocks via local zeitgebers such as nutrients and hormones [115]. A mismatch between the intrinsic circadian clock and behavior, as occurs during shift-work, leads to chronodisruption and is associated with several diseases including metabolic syndrome and obesity. A HFD alters the phase and amplitude of clock genes that regulate the circadian rhythm and contributes to chronodisruption [115,116]. In obese patients, the nocturnal rise in plasma ghrelin levels is blunted while the amplitude of the diurnal rhythm in leptin levels is increased [117]. GLP-1 levels peak during the day in humans, but the rhythmicity was also lost in obese patients [118]. These alterations in the dynamics of appetite-regulating hormones in obesity may alter the relationship among complex systems that regulate energy homeostasis. In humans intermittent fasting has gained popularity as a weight loss diet. Food is then only consumed within a consistent time window during the normal feeding period, thereby lengthening the daily fasting period. Enforcing nutrient utilization rhythms during chronodisruption leads to rhythmic activation of clock genes that amplify nutrient response mechanisms [119]. A recent randomized controlled trial in obese patients reported that time-restricted eating during eight weeks reduced body weight, insulin resistance and oxidative stress versus a control group that had no meal timing restrictions [120]. A similar randomized, isocaloric trial (five weeks) in men with prediabetes reported that time-restricted eating improved insulin sensitivity, blood pressure and oxidative stress even without weight loss in men [121]. It remains to be investigated whether intermittent fasting also results in a restoration of the gut hormone balance.

## 7. Strategies for the Management of Obesity: Role of Gut Hormones

### 7.1. Diet-Induced Weight Loss

Caloric restriction induces weight loss in obese individuals and restores the preprandial rise in ghrelin plasma levels [41]. Evidence from animal studies suggests that this increase in ghrelin levels may resensitize the brain and overcome ghrelin resistance to induce rebound weight gain [122]. Thus, ghrelin may act as a survival hormone to prevent further weight loss during a negative energy balance.

The effect of caloric restriction on plasma motilin levels has not been studied and has been hampered by the fact that motilin does not exist in rodents [29].

In obese patients, lower postprandial levels of GLP-1 and PYY were observed along with increased appetite scores, following an 8-week low-energy intake diet and a 2–3 week refeeding period [123]. Similarly, reductions in leptin, PYY and CCK were observed following a weight loss program with a very low energy diet which was accompanied by an increase in subjective appetite scores [124,125]. Importantly, one year after initial weight reduction, levels did not revert to levels recorded before weight loss, suggesting that alterations in gut hormone levels may facilitate regain of lost weight [124].

Interest in prebiotic supplementation with oligofructose or inulin for weight management stems from studies in rodents that reported reductions in body weight and altered gut hormone levels [126,127]. Prebiotic fibers are fermented by the gut microbiota to short chain fatty acids (SCFAs) that act on enteroendocrine cells via FFAR2 or FFAR3 to affect gut hormone release [106]. In a randomized, double-blind placebo controlled trial, oligofructose supplementation for 12 weeks reduced body weight in overweight and obese adults [128]. Ghrelin levels were reduced and PYY, but not GLP-1 levels were increased. Targeted delivery of the SCFA propionate to the colon of overweight patients with an inulin-propionate ester reduced energy intake and increased postprandial plasma PYY and GLP-1 levels in overweight patients [129]. Supplementation for 24 weeks reduced weight gain and prevented the deterioration in insulin sensitivity observed in the inulin control group. However, the rise in PYY and GLP-1 levels was not observed in the long-term study, indicating that desensitization may have occurred. A recent randomized clinical trial investigated the impact of modulation of the microbiome with isoenergetic diets that differed in their concentrations of prebiotics. The high-fiber diet selectively promoted a group of SCFA producers as the major active producers. When the SCFA producers were present in greater diversity and abundance, the improvement in haemoglobin A1c levels was greater, possibly reflecting in part increased GLP-1 production [130]. Evidence of crosstalk between the gut microbiome is also derived from studies with administration of *Akkermansia muciniphila*, known to prevent diet-induced obesity [131]. This commensal bacterium increased levels of 2-acylglycerols, endogenous cannabinoids, known to stimulate GLP-1 levels via GPR119 [132].

### 7.2. Roux-en-Y Gastric Bypass Surgery Restores the Gut Hormone Balance

A Roux-en-Y gastric bypass (RYGB) surgery, where the pouch of the stomach is bypassed to the small intestine, is an effective way of inducing and maintaining weight loss in morbidly obese patients. After RYGB surgery, the contact of nutrients with much of the stomach and duodenum is bypassed, resulting in a rapid delivery of undigested nutrients to the jejunum. This rerouting has been shown to affect the expression of nutrient sensors in the gut that together with other intestinal adaptations, such as changes in morphology and altered bacterial fermentation, contribute to alterations in gut hormone profiles [133,134,135].

Indeed, the reported weight loss with ensuing improvement in glucose homeostasis in patients undergoing RYGB surgery or sleeve gastrectomy is associated with elevated postprandial PYY and GLP-1 levels, even one year after surgery [136,137]. CCK-secreting cells are mainly located in the bypassed duodenum. In two studies, where the effect of RYGB on CCK was investigated, a faster and higher peak response towards a meal was found [137,138]. In addition, there is a possible association between the higher plasma levels of these satiety hormones and the reduced food reward system in patients after a RYGB surgery, these patients exhibit a modified behavioral and brain reward response to food [139,140].

**Figure 1:**
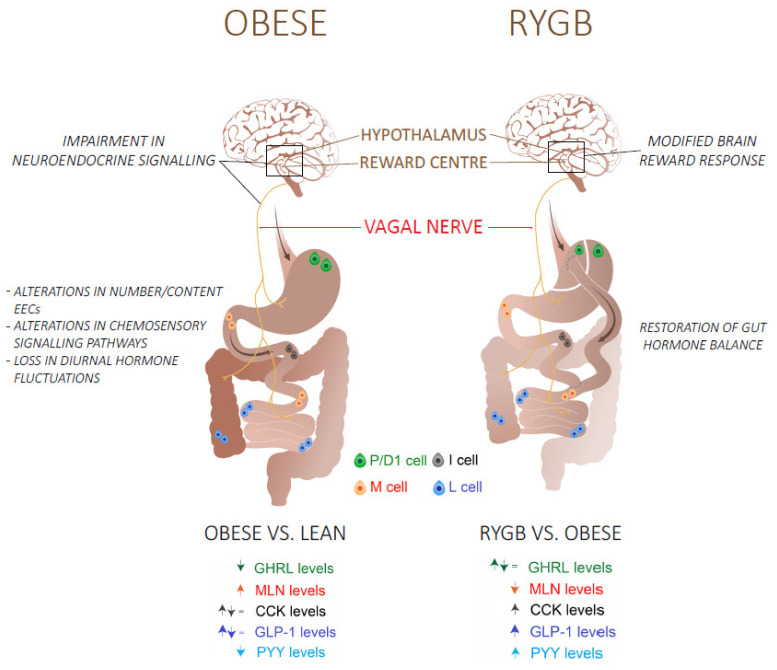
An overview of the mechanisms and the differences in fasting (GHRL, MLN) and postprandial (CCK, GLP-1, PYY) gut hormone plasma levels in obese/type 2 diabetes patients before and after a Roux-en-Y gastric bypass (RYGB) surgery. Abbreviations: GHRL: Ghrelin; MLN: Motilin; CCK: Cholecystokinin; GLP-1: glucagon-like peptide 1; peptide YY.

The reported effects of RYGB surgery on plasma ghrelin levels are inconsistent with a decrease, no change or an increase reported [136]. The size of the created pouch and difficulties inherent to the measurement of biological active octanoylated ghrelin levels have contributed to this. It is therefore unlikely that ghrelin is responsible for the post-surgical metabolic improvements. Regarding the other orexigenic hormone, motilin, Deloose et al. reported decreased motilin plasma levels in parallel with hedonic hunger scores after RYGB [44]. Figure 1 summarizes the differences in gut hormone levels in obese individuals before and after RYGB surgery.

### 7.3. Combination Therapy

GLP-1R agonists are used widely to treat T2DM. Liraglutide, which is administered once a day, was until now the only GLP-1 receptor (GLP-1R) agonist to be approved for weight management [141]. Recently, Semaglutide, a long acting GLP-1R agonist, has proven to be effective in weight management as an adjunct to lifestyle by inducing 14.9% weight loss from baseline in overweight and obese individuals [142]. Combined agonism, mostly by combining GLP-1 analogues with other food intake-inhibiting and/or glucose-lowering hormones, may cause a synergistic pharmacological action in obese individuals and patients with T2DM. Therefore, combination therapy is currently considered as the way to go to mimic the beneficial effects of RYGB surgery in a non-surgical manner [143]. Table 2 gives an overview of several combinations with GLP-1R analogues that are currently in clinical trial.

#### 7.3.1. GLP-1 and GIP

Glucose-dependent insulinotropic peptide (GIP) is an incretin hormone that is secreted by K-cells in response to nutrients to stimulate insulin secretion through activation of GIP receptors on pancreatic beta cells, and acts as a blood glucose stabilising hormone by regulating insulin and glucagon secretion [144,145]. GIP also exerts direct actions on lipid metabolism, promoting lipogenesis and weight gain, and GIPR agonists have been demonstrated to exacerbate the postprandial glucagon excursion in individuals with T2DM [146]. Therefore, GIP receptor (GIPR) antagonists were initially developed to induce weight loss and to control glycaemia levels in obesity and individuals with T2DM [147]. Even though individuals with T2DM have a decreased insulinotropic effect of GIP, due to impaired responsiveness by beta cells, the loss of GIP has been shown to enhance GLP-1R activity [55,148]. Evidence suggests that GIPR agonism can also positively impact body weight. A recent study showed that injection of a peripherally long acting, selective mouse GIPR agonist in DIO mice, lowered body weight due to reduced food intake [149]. Therefore, dual agonism of GLP-1R, which exerts glycaemic control, and GIPR represents a strategy in treating obesity and T2DM. Coadministration of the selective GIP receptor agonist, ZP4165, together with the GLP-1R agonist, liraglutide, in DIO mice resulted in superior body weight loss and improved blood glucose and plasma cholesterol levels [150]. Currently, tirzepatide, a dual-incretin peptide from Eli Lilly, has reached multi-dose clinical trials and shows promise in the treatment of obesity and T2DM [151].

#### 7.3.2. GLP-1 and GCG

The use of glucagon (GCG) with GLP-1 may intuitively appear contradictory since it antagonizes the effect of insulin and increases glucose levels, evoking hyperglycaemia. Nevertheless, glucagon also induces thermogenesis, increases energy expenditure and has hypolipidemic effects, which are beneficial for weight management in obese individuals [152]. Moreover, while chronic GCG stimulation exhibits glucose intolerance, acute GCG agonism at a lower dose, which is not able to evoke hyperglycaemia, enhances glucose tolerance and improves insulin sensitivity [153]. This suggests the use of GLP1-GCG dual agonists in not only obesity, but also in T2DM. Many preclinical studies have demonstrated the body weight and glucose lowering effects of GLP-1R/GCGR agonists. For example, a single high-dose or multiple low-dose injections of a GLP-1R/GCGR dual agonist induced body weight loss which was associated with increased energy expenditure and thermogenesis [154]. However, the effect of GLP-1R/GCGR dual agonists on body weight in human studies has not yet been found as effective as in animal studies. Cotadutide, a novel dual agonist by AstraZeneca, demonstrated superior results in body weight reduction relative to the GLP-1R agonist liraglutide during preclinical studies in DIO mice and normal weight cynomolgus monkeys [155]. Currently, results from Phase II clinical trials with cotadutide demonstrated beneficial effects on blood glucose levels, changes in liver fat and glycogen stores in patients with T2DM [156].

Oxyntomodulin (OXM) is a naturally occurring GLP1R/GCGR dual agonist that is secreted by L-cells after food intake to induce satiety and increase energy expenditure [157]. As native OXM has a very short half-life due to degradation by DPP4 and fast renal clearance, OXM analogues are being developed as a therapeutic candidate to treat obesity and T2DM. Recently, a PEGylated analogue showed a 27.1% body weight reduction at a high dose in DIO mice, which was significantly higher than the weight loss effect with liraglutide [158].

#### 7.3.3. GLP-1 and PYY_3-36_

The combination of GLP-1 analogue with PYY_3-36_ mainly has a role in body weight management. Co-infusion of PYY_3-36_ and GLP-1 reduced energy intake by 30% compared to placebo in overweight men, which was not achieved when a mono-infusion was administered of PYY_3-36_ or GLP-1 [159]. In addition, co-administration of PYY_3-36_ with oxyntomodulin reduced energy intake by 42.7% in overweight and obese volunteers, and the effect was more pronounced than when either hormone was infused separately [160]. No drugs are yet in clinical trials for combinations with PYY_3-36_.

#### 7.3.4. GLP-1, GCG and GIP

The combination of three gut hormones, triagonists, have emerged as new way of inducing multiple metabolic improvements. An acylated GLP-1R/GCGR/GIPR triagonist exerted in vivo and in vitro receptor activity in rodents with superior metabolic effects, improved glycaemic control and body weight loss, relative to their co-agonists [161]. HM15211 (Hanmi Pharmaceuticals) is a triagonist with high GCG activity for obesity treatment and a balanced GLP-1 and GIP activity, to neutralize the hyperglycemic risk of GCG. Preclinical studies with HM15211 have shown improved weight loss, reduced liver fat and possibly inflammation, and may be effective for the treatment of non-alcoholic fatty liver disease as well [162]. HM15211 is currently in phase II clinical trials with a 30% reduction of liver fat in comparison to placebo after a 12-month treatment [163].

Multi-agonists are the next generation of therapies to treat patients with T2DM and obesity. They avoid the adverse effects of surgery (malnutrition, post-prandial hypoglycaemia, bowel obstruction, etc.) and GLP1R agonists (gastrointestinal symptoms). Multi-agonists can therefore be a solution for these individuals as a way to manage body weight.

## 8. Conclusions

Gut hormones are important players in the regulation of appetite. Obesity has a clear impact on fasted and meal-related fluctuations in gut hormone release but the effect on some hormones remains controversial. The mechanisms involved are complex and multifactorial, relating to changes in the number/content of EECs, effect of age and gender, alterations in nutrients’ sensing mechanisms that regulate postprandial responses, alterations in diurnal fluctuations, and may also involve alterations in the central responsiveness to gut hormones. Further exploration of the crosstalk between the gut microbiome and EECs is of interest. Restoring the disordered gut hormone balance in obesity by targeting nutrient sensors in selective regions of the gut or by combined administration of gut peptide mimetics represent a major potential therapeutic targets to improve the prevention and management of obesity.

## Figures and Tables

**Table 1:** An overview of the meal-related fluctuations in gut hormone levels and their alterations in obesity and type 2 diabetes patients.

Hormone	Localisation	Meal-Related Fluctuations	Effect on Food Intake	Dysregulation in Obesity and Type 2 Diabetes			
				Release	↓	↑	=
Ghrelin (GHRL)	P/D1 cells (stomach)	Preprandial rise	Orexigenic	Fasting	[38,39,40,41,42,43]		
Motilin (MLN)	M cells (small intestine)	Preprandial rise	Orexigenic	Fasting		[44]	
Cholecystokinin (CCK)	I cells (small intestine)	Postprandial rise	Anorexigenic	Postprandial	[43,45]	[46]	[47]
Glucagon-like-peptide-1 (GLP-1)	L cells (small intestine)	Postprandial rise	Anorexigenic	Postprandial	[48,49,50,51,52]	[53]	[54,55,56]
Peptide-YY (PYY)	L cells (colon)	Postprandial rise	Anorexigenic	Postprandial	[43,57,58]		

**Table 2:** An overview of several combination therapies with GLP-1R agonists that are currently in clinical trials.

Combination Therapy	Physiological Effect	Drug Candidates		
GLP-1–GIP	Insulinotropic effect Decrease food intake cardiovascular protection	Drug	Company	Status
		Tirzepatide	Eli Lilly	Phase II
GLP-1–GCG	Insulinotropic effect cardiovascular protection Decrease food intake Increase energy expenditure	Drug	Company	Status
		Cotadutide	Astrazeneca	Phase II
		Efinopegdutide	Hanmi Pharmaceuticals	Phase II
GLP-1–GCG-GIP	Insulinotropic effect Increase energy expenditure cardiovascular protection Decrease food intake	Drug	Company	Status
		MAR423	Novo-nordisk/Marcadia	Phase I
		HM15211	Hanmi Pharmacueticals	Phase II

Glucagon-like-peptide 1 (GLP-1), glucose-dependent insulinotropic peptide (GIP), glucagon (GCG).
